# Recent discoveries of new *Elephantomyia* (Diptera, Limoniidae) fossils in Baltic amber

**DOI:** 10.1038/s41598-021-03022-3

**Published:** 2021-12-08

**Authors:** Iwona Kania-Kłosok, Wiesław Krzemiński

**Affiliations:** 1 Department of Biology, Institute of Biology and Biotechnology, University of Rzeszów, Rzeszów, Poland; 2 Institute of Systematics and Evolution of Animals, Polish Academy of Sciences, Krakow, Poland

**Keywords:** Evolution, Zoology

## Abstract

New data on the genus *Elephantomyia* (Diptera: Limoniidae) from Baltic amber are presented. A new subgenus *Hoffeinsonia* subgen. nov. is established with one new species: *Elephantomyia* (*Hoffeinsonia*) *prima* sp. nov. The new subgenus is characterized by a wing at most 2.5 × as long as it is wide without a darker pattern along the veins Sc and R_1_, elongate Sc, straight vein R_1_, sharp half of vein R_2+3+4_ sharply arched to the upper edge of the wing, short, wide, trapezoidal d-cell and oval pterostigma. The fossil subgenus *Hoffeinsonia* subgen. nov. shares features with the extant subgenera *Elephantomyodes* and *Elephantomyia*. One other extinct species of *Elephantomyia* was discovered and described herein as *E.* (s. str.) *christelae* sp. nov. Such features as a very elongate vein R_2+3+4_, 2.5 × as long as the Rs easily allowing this new species to be distinguished from the other fossil representatives of the genus *Elephantomyia*. The taxonomic decision on *Elephantomyia grata* as a species placed in nominative subgenus is provided. A list and key of fossil species of *Elephantomyia* are given. The morphological pattern of the genus is discussed in relation to the adaptation to a specific food spectrum, coevolution with Angiospermae of the representative genus *Helius* known since Cretaceous and closely related to this genus representatives of the much younger genus *Elephantomyia*.

## Introduction

The genus *Elephantomyia* Osten Sacken^1^, comprises over 140 extant species, which occur mainly in Neotropical and Afrotropical regions. In the Neotropics, the genus *Elephantomyia* is represented by 38 species, which belongs to three subgenera: the typical subgenus *Elephantomyia*, subgenus *Elephantomyina* Alexander^2^ (one species) and the subgenus *Xenoelephantomyia* Alexander^3^ (one species). In the Afrotropics, the genus *Elephantomyia* is represented by over 30 species, which are classified to only one subgenus *Elephantomyia*. The subgenus *Elephantomyodes* Alexander^4^ was recorded from Holarctic (only two species), from China /Hainan, Taiwan/, India /Tamil Nadu, Assam/, Malaysia /Borneo/, Indonesia/Java, Sumatra, Flores, Sulawesi/, Philippines, Thailand) (18 species) and Australian/Oceanian region (13 species)^5^.

**Figure 1:**
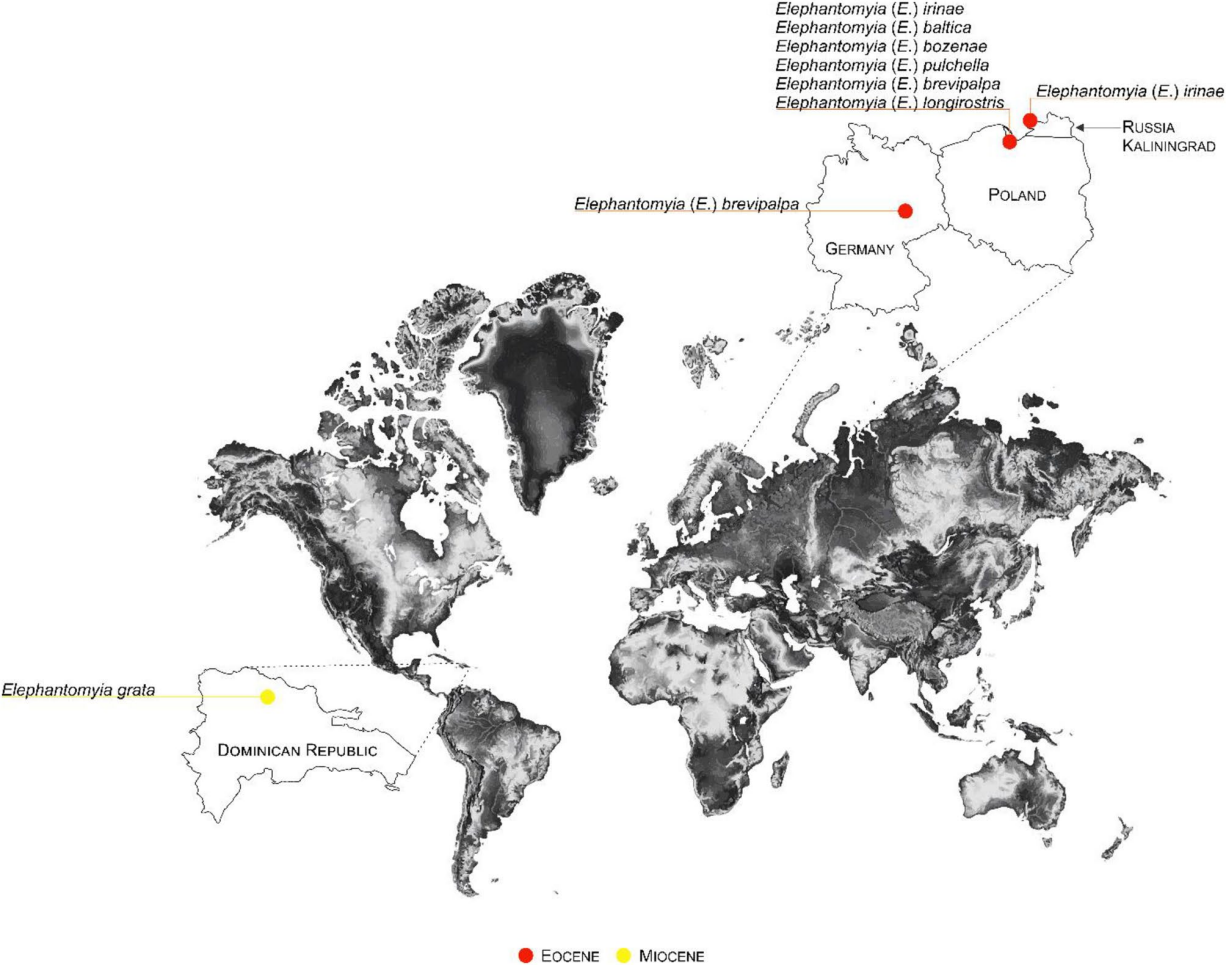
Map with enlarged view of the distribution of know localities of the representatives of the genus *Elephantomyia*. Map was built using the map Maps-For-Free (https:// maps- for- free. com) and modified with the software programs Corel Draw and Corel Photopaint X7.

From fossil record seven species of *Elephantomyia* are known, six of them are known to have come from Eocene Baltic amber and belong to nominative subgenus *Elephantomyia*^6–8^*.* We only know of one species of *Elephantomyia* from Miocene to this day unclassified to any subgenus—*Elephantomyia grata* Podenas & Poinar^9^ (Table 1; Fig. 1). None of the other three subgenera of *Elephantomyia*: *Elephantomyina*, *Elephantomyodes*, *Xenoelephantomyia* have so far been found in the fossil record.

**Table 1:** List of fossil species belonging to the genus *Elephantomyia* described before the present study, with age and localities.

Species	Age	Locality
*Elephantomyia* (s. str.) *grata* Podenas & Poinar^9^	Miocene	Dominican amber
*Elephantomyia* (s. str.) *baltica* Alexander^6^	Eocene	Baltic amber
*Elephantomyia* (s. str.) *bozenae* Kania^8^	Eocene	Baltic amber
*Elephantomyia* (s. str.) *brevipalpa* Loew^7^	Eocene	Baltic amber
*Elephantomyia* (s. str.) *irinae* Kania^8^	Eocene	Baltic amber
*Elephantomyia* (s. str.) *longirostris* Loew^7^	Eocene	Baltic amber
*Elephantomyia* (s. str.) *pulchella* Loew^7^	Eocene	Baltic amber

Baltic amber forms the largest amber deposit in the world and is a relevant source for fossil insects. Diptera are largely dominant and diverse among animal inclusions in this kind of resin what may be related to the fact that the deposits of the Eocene Baltic amber were formed over a relatively long time and under various environmental conditions^10^.

The new materials under investigation made it possible to discover a representative of a new subgenus of *Elephantomyia* in the fossil material. This new, peculiar discovery from Baltic amber described herein, provides evidence of the existence of craneflies belonging to a new subgenus in the Eocene and this is the first case to confirm the existence of a subgenus other than nominative the *Elephantomyia* in the past.

## Results

### Systematic palaeontology

Order Diptera Linnaeus^11^.

Infraorder Tipulomorpha Rohdendorf^12^.

Family Limoniidae Speiser^13^.

Subfamily Limoniinae Speiser^13^.

Genus *Elephantomyia* Osten Sacken^1^.

Type species: *Limnobiorhynchus canadensis* Westwood^14^: 684, sensu Osten Sacken^1^: 221; by original designation (= *westwoodi* Osten Sacken^15^: 109, misidentification).

***Elephantomyia*** Osten Sacken^1^ subg. *Elephantomyia* Alexander, 1938 emend.

*Emended diagnosis* Wing at most 2.5 × as long as wide without darker pattern along vein Sc and R_1_; pterostigma oval; tip of Sc before or at Mb bifurcation level, opposite crossvein m-cu; vein R_1_ slightly curved at the tip, vein R_2+3+4_ slightly arched to the upper edge of wing, the distance between veins R_2+3+4_ and R_1_ and between veins R_2+3+4_ and R_5_ comparable; d-cell almost rectangular; two anal veins well developed; gonostyles elongate, approximately 1/3 the length of gonocoxite or longer, gonocoxites elongate but rather wide, approximately twice their width or shorter.

***Elephantomyia* (*Elephantomyia*) *grata*** Podenas & Poinar^9^.

*Elephantomyia grata*: Podenas & Poinar^9^: 867.

*Remark* The species *Elephantomyia grata* Podenas & Poinar^9^ described from Dominican amber has not been classified into a subgenus so far. Based on species characteristics consistent with the diagnosis of the subgenus, we decided to classify *E. grata* under the subgenus *Elephantomyia*. *E. grata* subgeneric placement is based on the original description without examination of the type specimen.

**Figure 2:**
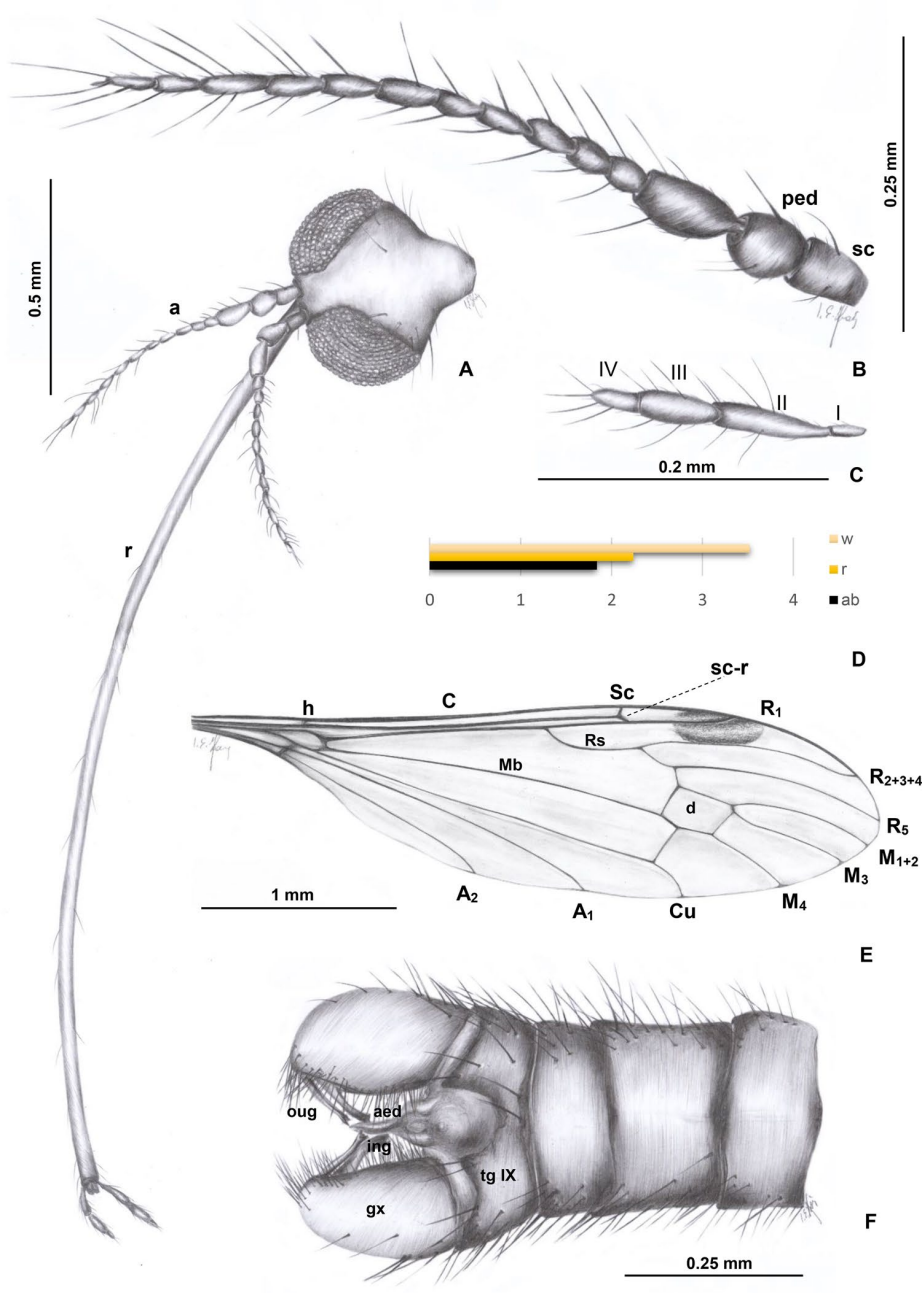
*Elephantomyia* (s. str.) *christelae* sp. nov. No. CCHH 874–2 (male), coll. Ch. & H. W. Hoffeins, holotype, drawings: (**A**) head (ventral view); (**B**) antenna; (**C**) palpus; (**D**) the diagram illustrating the relationship between the length of wing (w), rostrum (r) and abdomen (ab); (**E**) wing; (**F**) hypopygium (dorsal view). Abbreviation: a—antenna; r—rostrum; ped—pedicel; scp—scape; I-IV—palpomeres I-IV; gx—gonocoxite.

**Figure 3:**
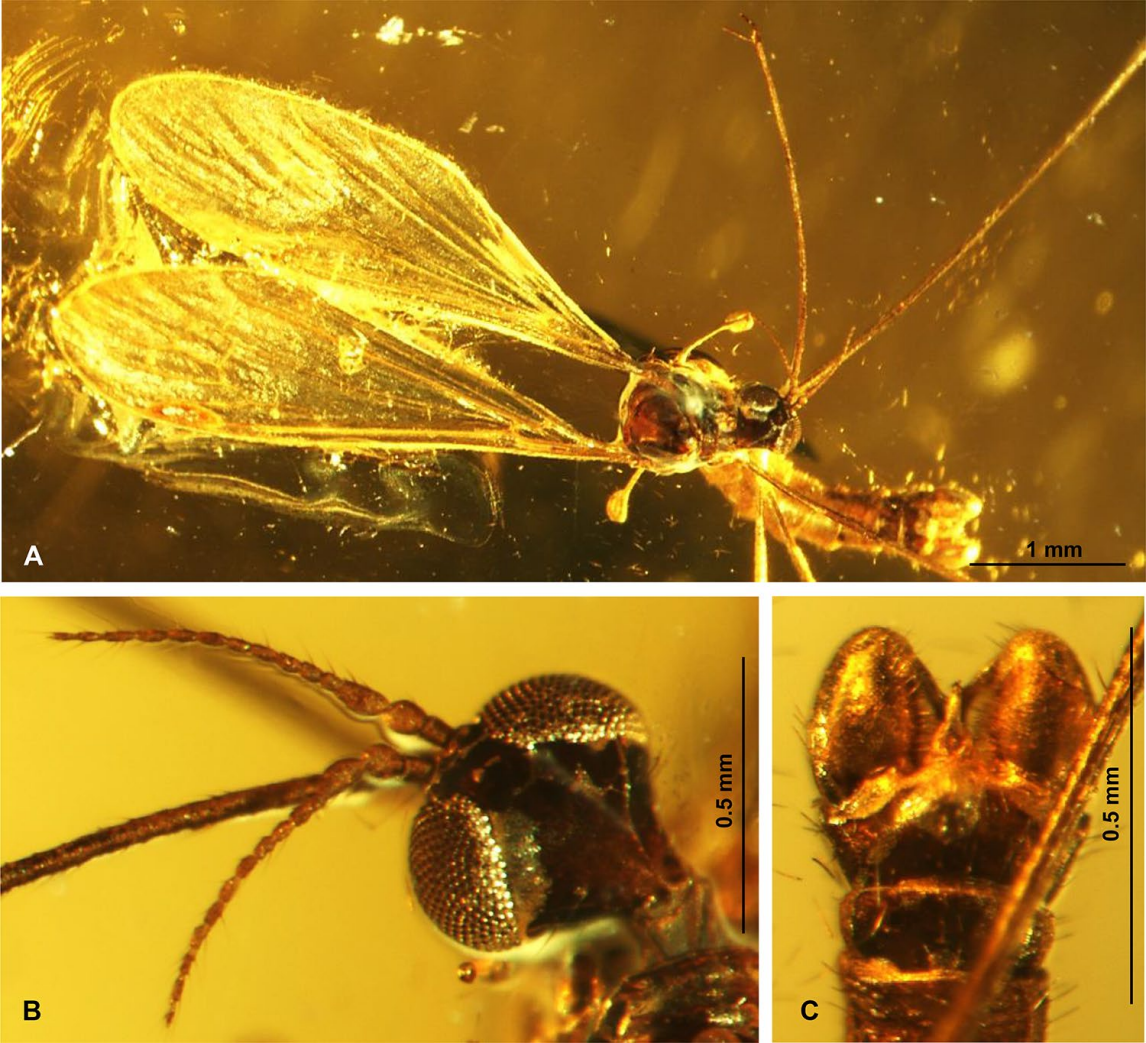
*Elephantomyia* (s. str.) *christelae* sp. nov. No. CCHH 874–2 (male), coll. Ch. & H. W. Hoffeins, holotype, photographs: (**A**) the body (dorso-ventral view); (**B**) enlarged view of antenna; (**C**) hypopygium.

**Figure 4:**
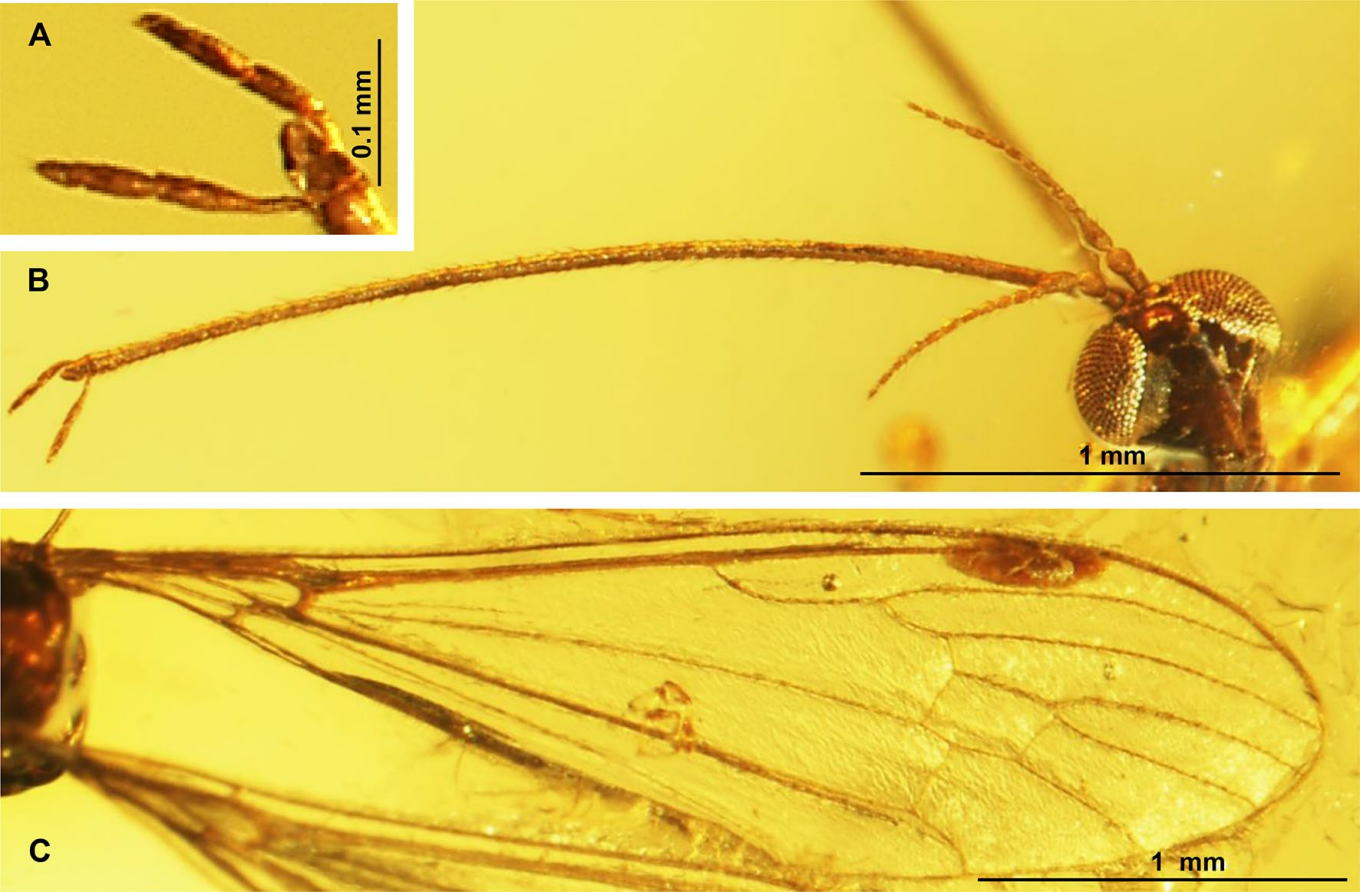
*Elephantomyia* (s. str.) *christelae* sp. nov. No. CCHH 874–2 (male), coll. Ch. & H. W. Hoffeins, holotype, photographs: (**A**) palpi; (**B**) head; (**C**) wing.

***Elephantomyia* (*Elephantomyia*) *christelae*** sp. nov. (Figs. 2, 3 and 4).

*Diagnosis* Antennae 14-segmented; rostrum shorter than wing, only slightly longer than 1/2 of wing, longer than abdomen; palpus longer than glossal lobes; R_2+3+4_ very elongate, 2.5 × as long as Rs; d-cell short and wide, 1.5 × as long as wide; m-cu just before half the length of d-cell; M_3_ 2 × longer than d-cell; vein m-m very short, almost completely reduced; vein Rs relatively short, length of vein Rs arranges only about twice the length of the basal deflection of R_5_; shorter than R_2+3+4_.

*Etymology* The new species is dedicated to Christel Hoffeins from Hamburg, Germany, the amber collection owner and expert of the Baltic amber inclusions.

*Material examined* Holotype No. CCHH 874–2 (male), coll. Ch. & H. W. Hoffeins, the specimen housed in Senckenberg Deutsches Entomologisches Institut (SDEI) Müncheberg, Germany.

*Horizon and locality* The age of Baltic amber has been a matter of debate for many years^9,16–23^. But, the most current state of knowledge is that it is of Priabonian age^17^. This means it is between 38 and 34 million years old (based on pollen, spores and phytoplankton of the amber embedding layer, the Blue Earth). The age of Baltic amber has also been estimated to approximately 47–41 Ma, which is mainly based on a study by Ritzkowski^24^; however, the reliability of the methods used in his study have been questioned due to contaminations that can lead to older age estimations^16,17^. The age range of all Baltic amber bearing strata possibly cover 48 to 23 million years, and it is still debatable^18–23^).

*Description.* Body (Fig. 3A): brown with dark distal part of abdomen darker than rest of body, body 3.28 mm long (without rostrum).

Head (Figs. 2A, 3A, 4B): 0.45 mm wide, 0.34 mm high; rostrum elongate 2.24 mm long, shorter than wing, terminate just behind half of wing, rostrum longer than abdomen (1.84 mm long) (Figs. 2D, 3A, 4B); antenna (Figs. 2A, B, 3A, B, 4B) small, 0.64 mm long, flagellar segments crowded, scape cylindrical, widened, pedicel wide, first flagellomere elongate, second flagellomere only slightly shorter than rest of flagellomeres, flagellomeres 1–8 with two elongate setae on each flagellomere; flagellomeres 9–12 with four elongate setae.

Palpus (Figs. 2C, 4A, B): longer than glossal lobes, 0.19 mm long, 4-segmented, last palpomeres short, other palpomeres elongate, third palpomere shorter than second. The small microtrichia on all segments well visible.

Wing (Figs. 2E, 3A, 4C): 3.52 mm long, 0.88 mm wide; pterostigma present, darkened, oval, brown; vein Sc elongate, ending opposite 3/4 the length of Rs; sc-r short, one time the distance from the tip of Sc; vein Rs slightly arcuated, R_1_ ending before half of the length of R_2+3+4_; r–r (R_2_) atrophy; d-cell 0.26 mm long, M_3_ 0.61 mm long; A_1_ almost straight, A_2_ slightly curved at wing margin.

Hypopygium (Figs. 2F, 3A, C): 0.46 mm, gonocoxite approximately two times as long as wide with elongate and narrow, lobe-shaped interbase; male genitalia with lobe, outer gonostylus (branch II = clasper of gonostylus; ventral gonostylus) narrow, distinctly bifid at the end, the distal part slightly curved outside; inner gonostylus (branch I = lobe of gonostylus; dorsal gonostylus) slightly widened at base, directed inside of hypopygium.

*Remarks* A well preserved holotype specimen, but only partially preserved legs.

*Comparison* The species differ from all other known from fossil records due to the very short cross-vein m-m. Veins M_1+2_ and M_3_ in *E.* (*E.*) *christelae* sp. nov. is narrowly separated and the distance between veins M_1+2_/M_3_ is smaller than between veins M_3_/M_4_ while in other known fossil record species of *Elephantomyia* veins M_1+2_ and M_3_ are widely separated and the distance between veins M_1+2_/M_3_ and veins M_3_/M_4_ is comparable, cross-vein m-m is well developed. Moreover, this species is characterized by the occurrence of 15-segmented antenna while in *E.* (*E.*) *baltica* and *E.* (*E.*) *brevipalpa* antennae are 14-segmented. Palpus in *E.* (*E.*) *christelae* sp. nov. is elongate, longer than glossal lobes, while in *E.* (*E.*) *brevipalpa* palpus is very short, being less than half the length of the rostrum’s glossal lobes. Vein Rs in *E.* (*E.*) *brevipalpa* is as long as, or longer than vein R_2+3+4_, in contrast to *E.* (*E.*) *christelae* sp. nov. where Rs is distinctly shorter than R_2+3+4_. *E.* (*E.*) *christelae* sp. nov. differs also from the other species of *Elephantomyia* in the ratio between wing, rostrum, and abdomen length. In *E.* (*E.*) *christelae* sp. nov. rostrum is slightly longer than the abdomen, being longer than half wing length, but shorter than wing.

***Hoffeinsonia*** subgen. nov.

*Type species: Elephantomyia* (*Hoffeinsonia*) *prima* subgen. et sp. nov.

*Diagnosis* Wing at most 2.5 × as long as wide without darker pattern along vein Sc and R_1_; pterostigma oval; vein Sc elongate, tip of Sc beyond Mb bifurcation level, opposite crossvein m-cu; vein R_1_ straight, basal half of vein R_2+3+4_ sharpy arched to the upper edge of wing, veins R_2+3+4_ and R_1_ runing closer together than veins R_2+3+4_ and R_5_; d-cell short, wide, trapezoidal; two anal veins well developed; gonostyles small, about 1/3 length of gonocoxite, gonocoxites elongate and rather narrow, longer than twice their width.

*Etymology* The new subgenus is dedicated to Christel Hoffeins from Hamburg, Germany, the amber collection owner and expert of the Baltic amber inclusions.

*Description* As for species.

*Comparison* What occurs in *Elephantomyina* is a strong supernumerary cross-vein connecting vein R_2+3+4_ shortly before tip of latter, cross-vein r-m connecting with Rs a short distance before its fork^2^, while in other subgenera of the genus *Elephantomyia* (including *Hoffeinsonia* subgen. nov.) the supernumerary cross-vein does not occur and cross-vein r-m connecting with Rs beyond of Rs. Moreover, as mentioned by Osten Sacken^25^: „anal field of wing reduced in area, with a single vein”, while in three other known subgenera of *Elephantomyia* and *Hoffeinsonia* subgen. nov. two well developed annal veins are observed.

Only the hind leg with part of the tarsus of the specimen of *Hoffeinsonia* subgen. nov. is preserved, but is clearly visible that basal and middle part of femur and part of tibia are pale. This feature and others such as: vein R_1_ straight, basal half of vein R_2+3+4_ sharply arched to the upper edge of wing, veins R_2+3+4_ and R_1_ running closer together than veins R_2+3+4_ and R_5_, reduced palpi are similar to these which occur in subgenus *Elephantomyodes*. The afore mentioned features are well visible in recent representatives of the subgenus as *Elephantomyia* (*Elephantomyodes*) *tianmushana* Zhang, Li and Yang^26^, *Elephantomyia* (*Elephantomyodes*) *sophiarum* Ito^27^, *Elephantomyia* (*Elephantomyodes*) *angusticellula* Alexander^28^ or *Elephantomyia* (*Elephantomyodes*) *major major* Alexander^4^. Pterostigma in *Hoffeinsonia* subgen. nov. is distinctly oval, like in *Elephantomyia*. The wing of *Hoffeinsonia* subgen. nov. is wider than the wing of *Elephantomyodes* and arrange at most 2.5 × the length of its width, while the wing of *Elephantomyodes* 3.5x. Moreover, in *Elephantomyodes* along vein Sc and R_1_ occur darker pattern, in contrast to *Hoffeinsonia* subgen. nov.

**Figure 5:**
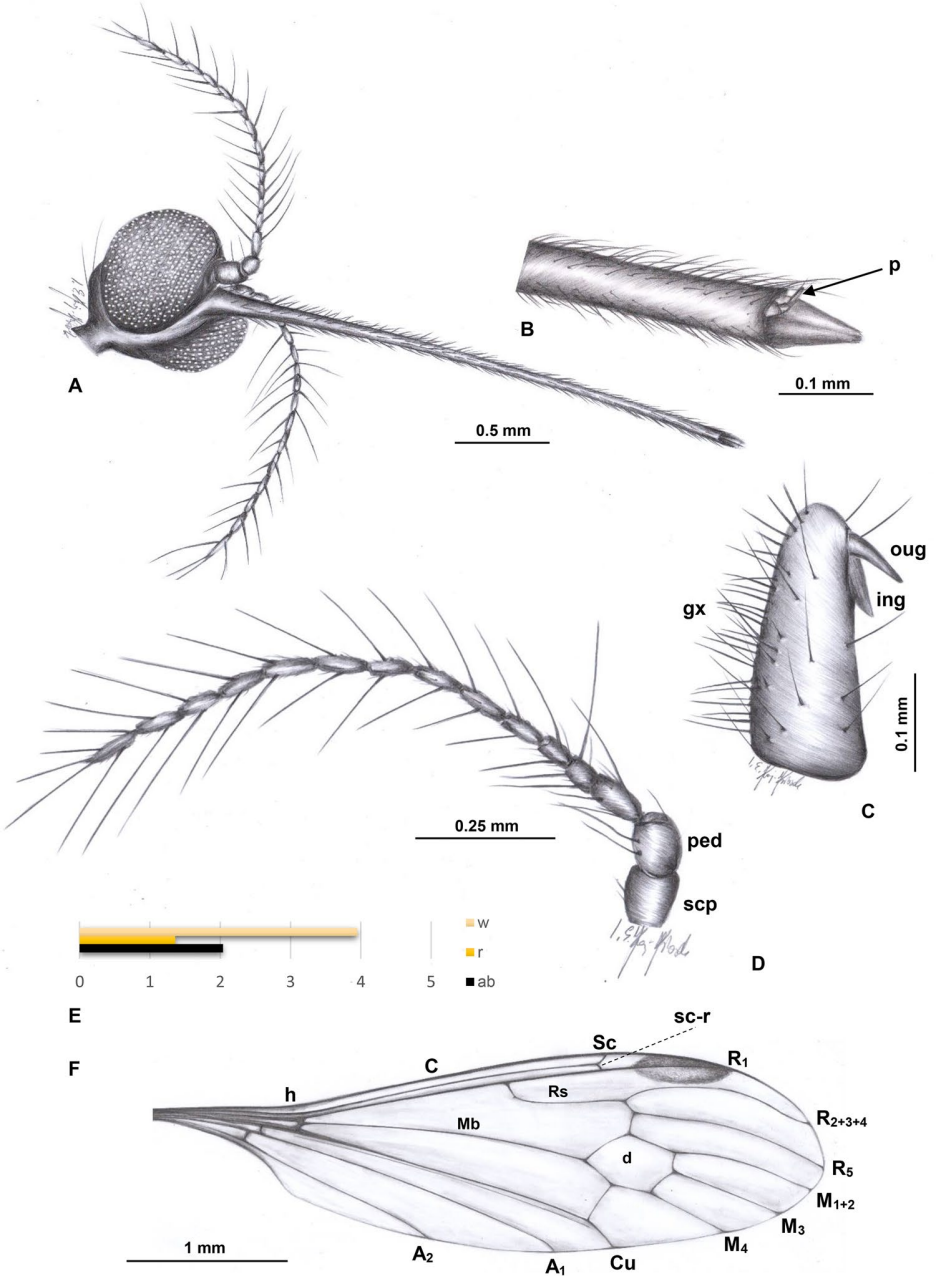
*Elephantomyia* (*Hoffeinsonia*) *prima* sp. nov. No. CCHH 874–1 (male), coll. Ch. & H. W. Hoffeins, holotype, drawings: (**A**) head, latero-ventral view; (**B**) enlarged view of apical part of rostrum; (**C**) gonocoxite and gonostyles; (**D**) antenna; (**E**) the diagram illustrating the relationship between the length of wing (w), rostrum (r) and abdomen (ab); (**F**) wing. Abbreviation: a—antenna; ped—pedicel; scp—scape; I–IV—palpomeres I–IV; gx—gonocoxite; oug—outer gonostylus; ing—inner gonostylus; p—palpus.

**Figure 6:**
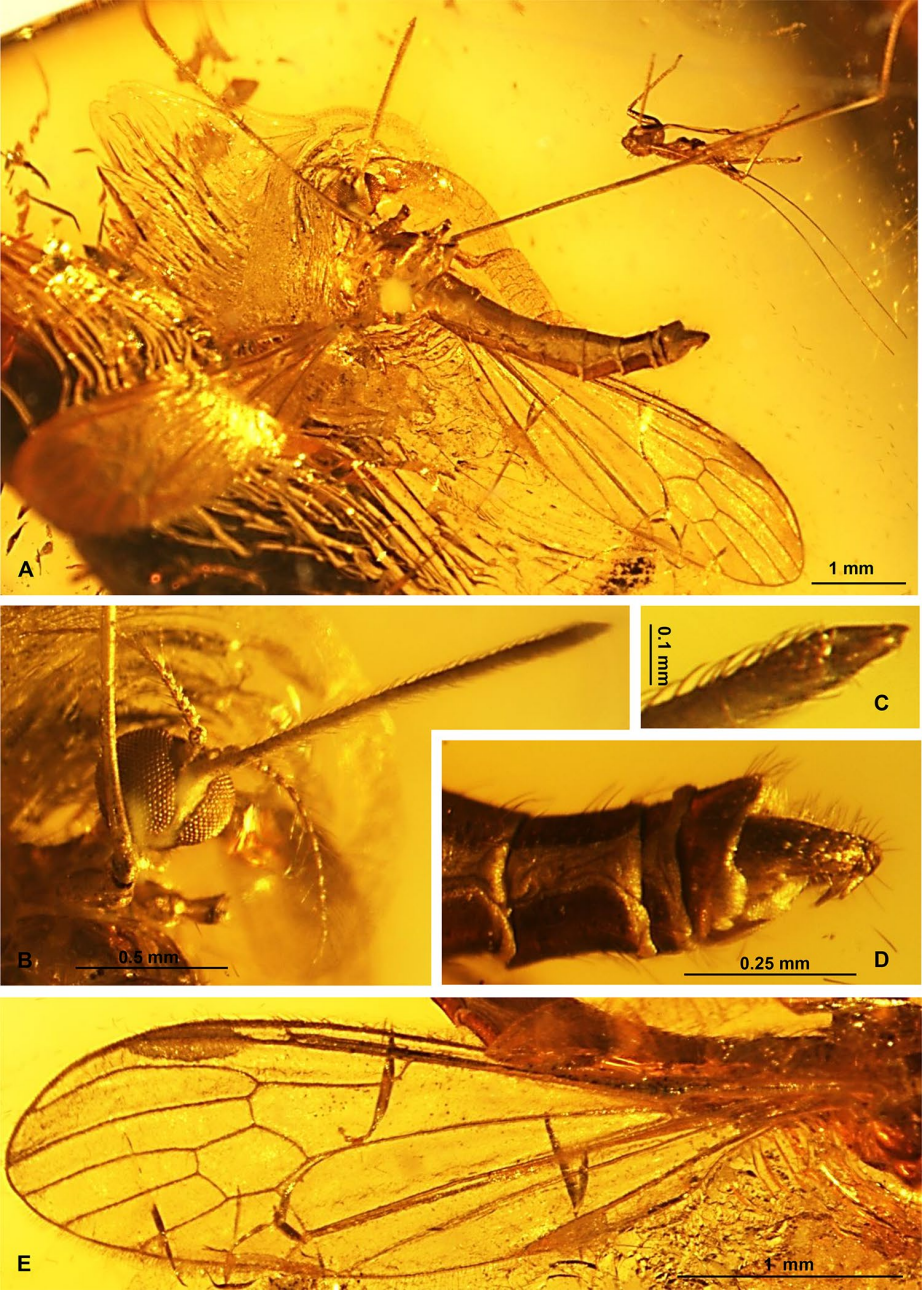
*Elephantomyia* (*Hoffeinsonia*) *prima* sp. nov. No. CCHH 874–1 (male), coll. Ch. & H. W. Hoffeins, holotype, photographs: (**A**) body, latero-dorsal view; (**B**) head, latero-ventral view; (**C**) apical part of rostrum with palpi visible; (**D**) hypopygium, lateral view; (**E**) wing.

**Figure 7:**
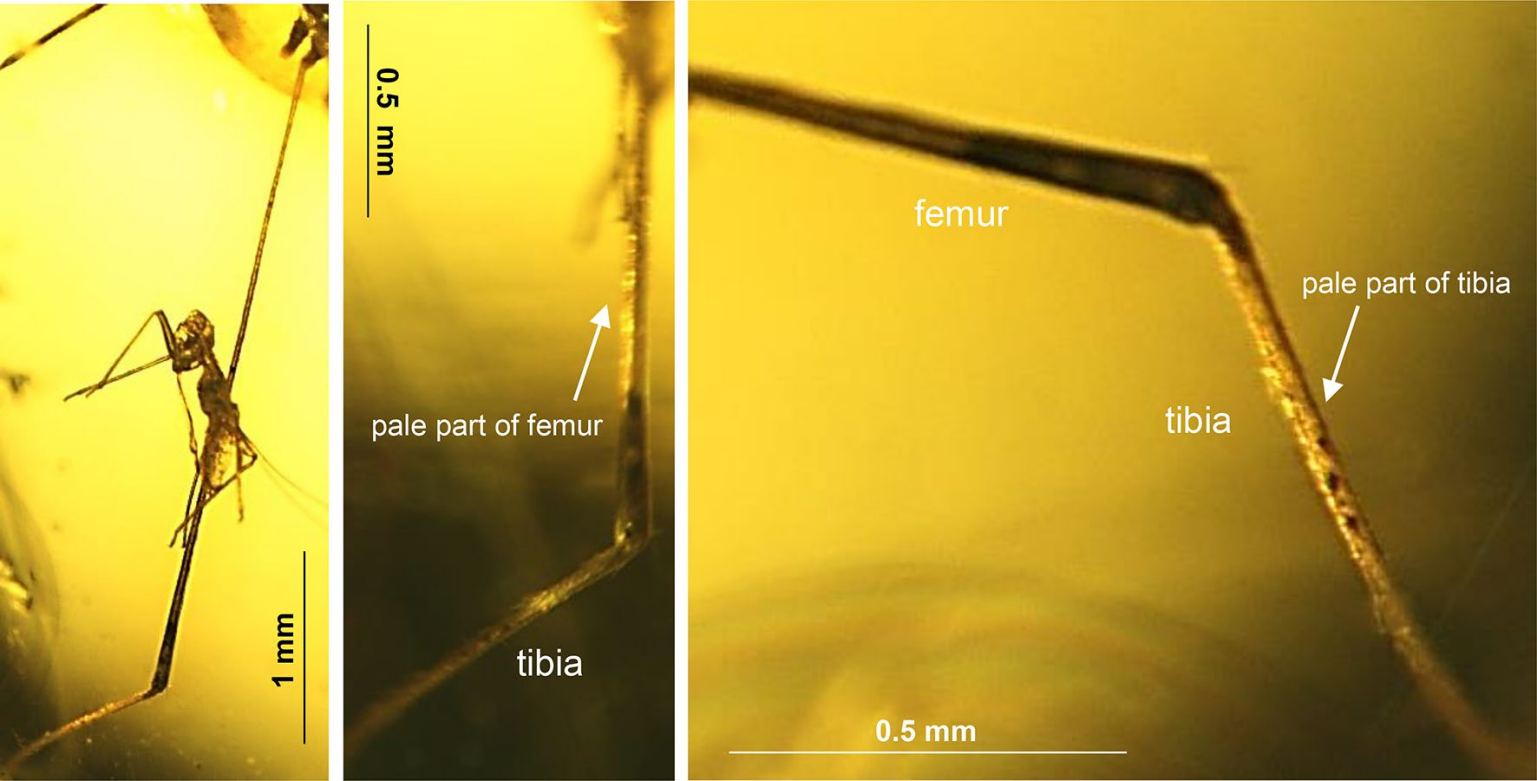
*Elephantomyia* (*Hoffeinsonia*) *prima* sp. nov. No. CCHH 874–1 (male), coll. Ch. & H. W. Hoffeins, holotype, photographs: (**A**) femur and part of tibia of hind leg; (**B**) pale parts of femur and tibia of hind leg visible; (**C**) enlarged view of pale part of hind tibia.

***Elephantomyia*** **(*Hoffeinsonia*)** ***prima*** subgen. et sp. nov. (Figs. 5, 6, and 7).

*Diagnosis* Antennae 15-segmented; rostrum shorter than wing, shorter than 1/2 of wing, shorter than abdomen; palpus shorter than glossal lobes; R_2+3+4_ 1.5 × as long as Rs; d-cell approximately 1.5 × as long as wide; m-cu in half the length of d-cell; M_3_ 1.5 × longer than d-cell; vein m-m well developed; length of vein Rs arranges only about five the length of the basal deflection of R_5_; R_5_ approximately as long as R_2+3+4_.

*Etymology.* The specific epithet is derived from „prima” (Latin) = the first.

*Material examined.* Holotype No. CCHH 874–1 (male), coll. Ch. & H. W. Hoffeins, the specimen housed in Senckenberg Deutsches Entomologisches Institut (SDEI) Müncheberg, Germany.

*Horizon and locality* as for *E.* (s.str*.*) *christelae* sp. nov.

*Description* Body 3.61 mm long (Fig. 6A).

Head (Fig. 6B, C) width 0.42 mm, 0.38 mm high; rostrum 1.36 mm long, approximately as long as half the body length, shorter than half wing; length of antenna 0.72 mm (Fig. 5A); scape elongate, cylindrical, pedicel oval, wider than scape and other antennal segments, flagellomeres 1 and 2 only slightly elongate, longer than wide, flagellomeres 3–15 elongate, longer than twice of its width with very elongate setae, approximately twice as long as its width or longer; length of flagellomeres: 1/0.08 mm; 2/0.06 mm; 15/0.04 mm; palpus very short, 0.13 mm long, shorter than glossal lobes.

Thorax (Fig. 6A): wing (Figs. 5B, 6A, E) longer than body, without colour pattern, 3.95 mm long, 1.10 mm wide; oval pterostigma brown, vein Sc elongate, tip of Sc beyond Mb bifurcation level, opposite crossvein m-cu; cross-vein sc-r one of its length from the tip of Sc, fork of Rb beyond half the length of wing towards the apex of wing; fork of Rs beyond fork of Mb level and opposite approximately 1/3 of d-cell length measured from fork of Mb; Rs 0.70 mm long; R_2+3+4_ approximately 1.6 × as long as Rs; cross-vein m-cu in half the length of d-cell; d-cell 0.42 mm long, wide, approximately 1.5 × as long as wide; length of M_3_ 0.64 mm; tip of A_1_ beyond fork of Mb toward the apex of wing; macrotrichia on radial and medial veins occur, vein M_3_ with macrotrichia arranged close to each other at equal intervals; hind legs with pale parts of femur and tibia (Fig. 7A–C).

Abdomen (Fig. 6A) 2.04 mm long; hypopygium 0.37 mm long with lobe—short, small, comparable length gonostyles, outer gonostylus (branch II = clasper of gonostylus; dorsal gonostylus) narrow, tappered at the end, strongly sclerotized, inner gonostylus (branch II = lobe of gonostylus; dorsal gonostylus) widened at the base and in its middle, narrowed at the end (Fig. 6D), less sclerotized than the outer gonostylus.

*Remarks* Well preserved holotype specimen, but only partially preserved legs.

Key to fossil species of the genus *Elephantomyia.*

**Figure 8:**
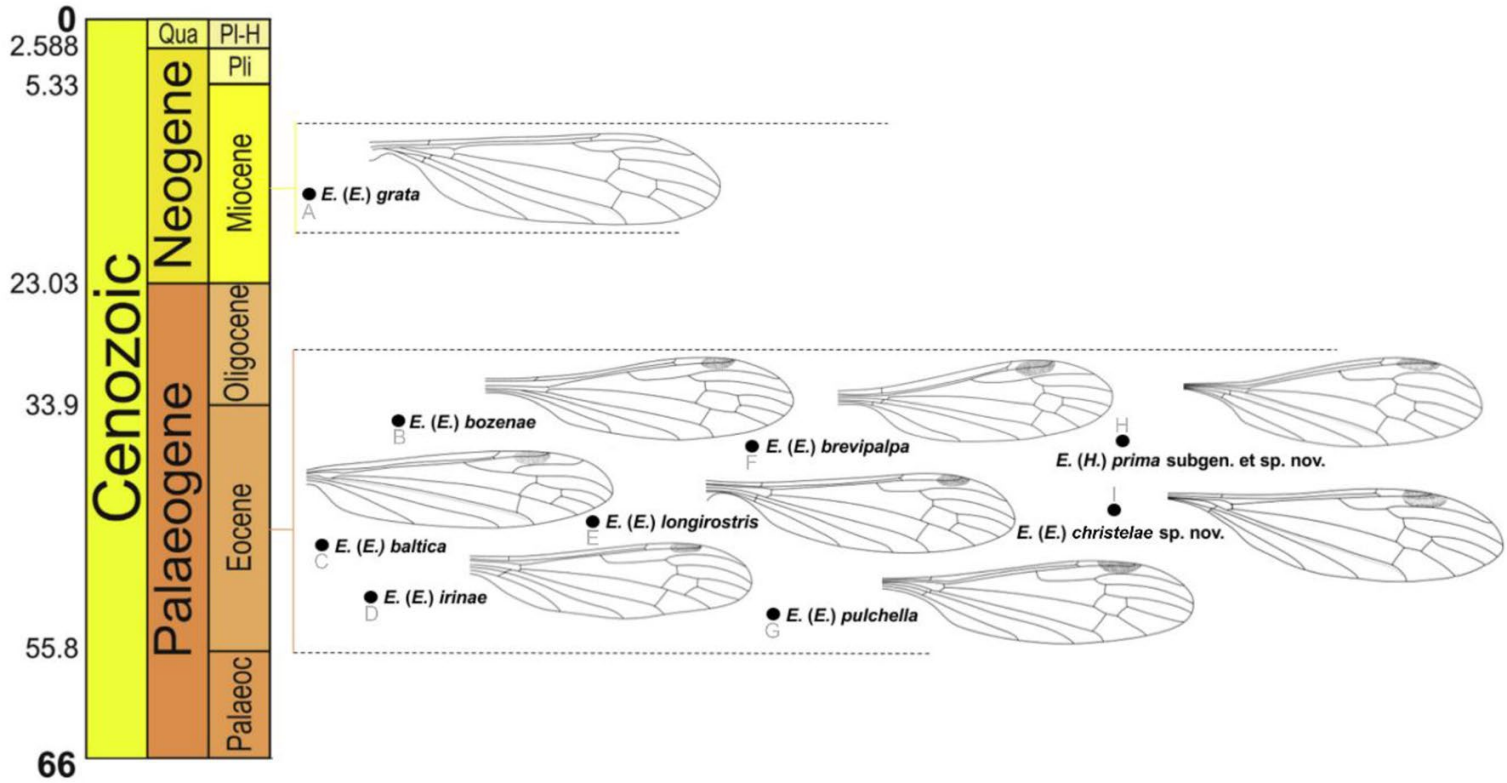
Chronostratigraphic distribution of *Elephantomyia*, fossil species, wings redrawn after Podenas and Poinar^9^; Kania^8^. Stratigraphic chart according to International Stratigraphic Chart, International Commission of Stratigraphy (v. 2021/05) https://stratigraphy.org/ chart (accessed on 16 September 2021).

**Figure 9:**
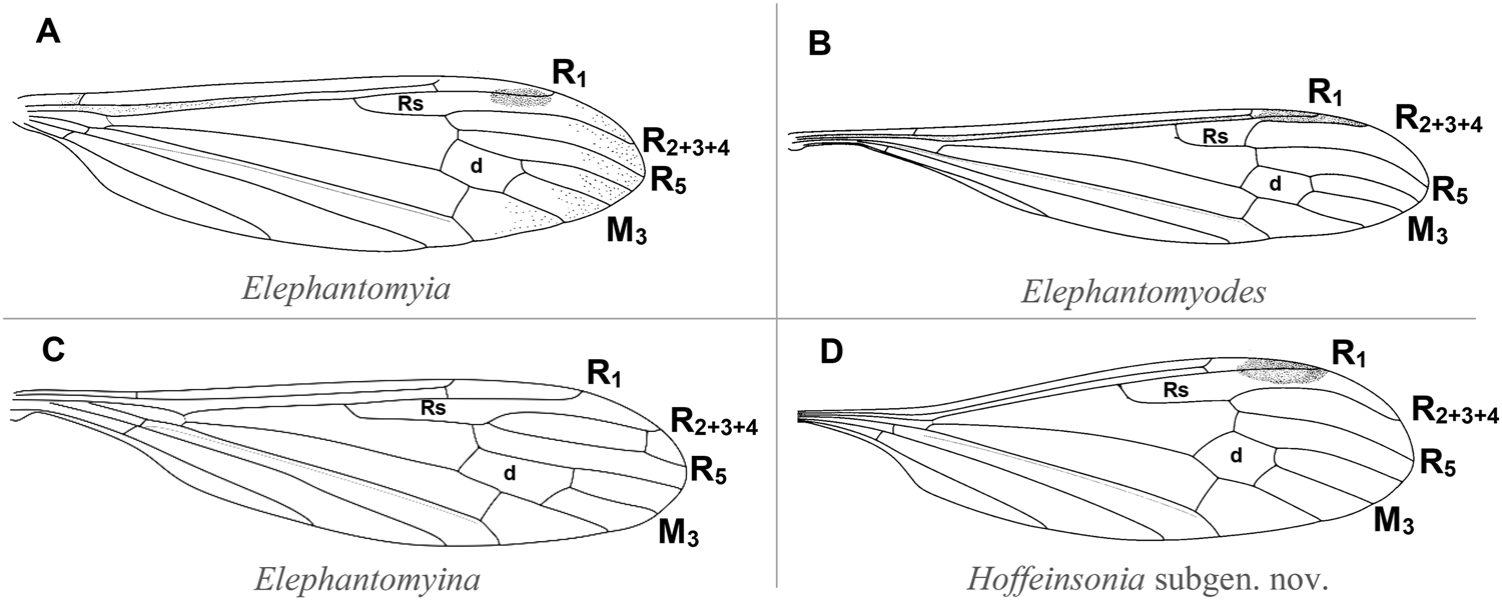
Wing venation of species belonging to different subgenera of genus *Elephantomyia*: (**A**) *Elephantomyia* (s. str.) *edwardsi* Lackschewitz^35^ (redrawn^36^); (**B**) *Elephantomyia* (*Elephantomyodes*) *major major* Alexander^4^ (redrawn^17^); (**C**) *Elephantomyia* (*Elephantomyina*) *supernumeraria* Alexander^37^ (redrawn^2^); (**D**) *Elephantomyia* (*Hoffeinsonia*) *prima* subgen. and sp. nov.

**Figure 10:**
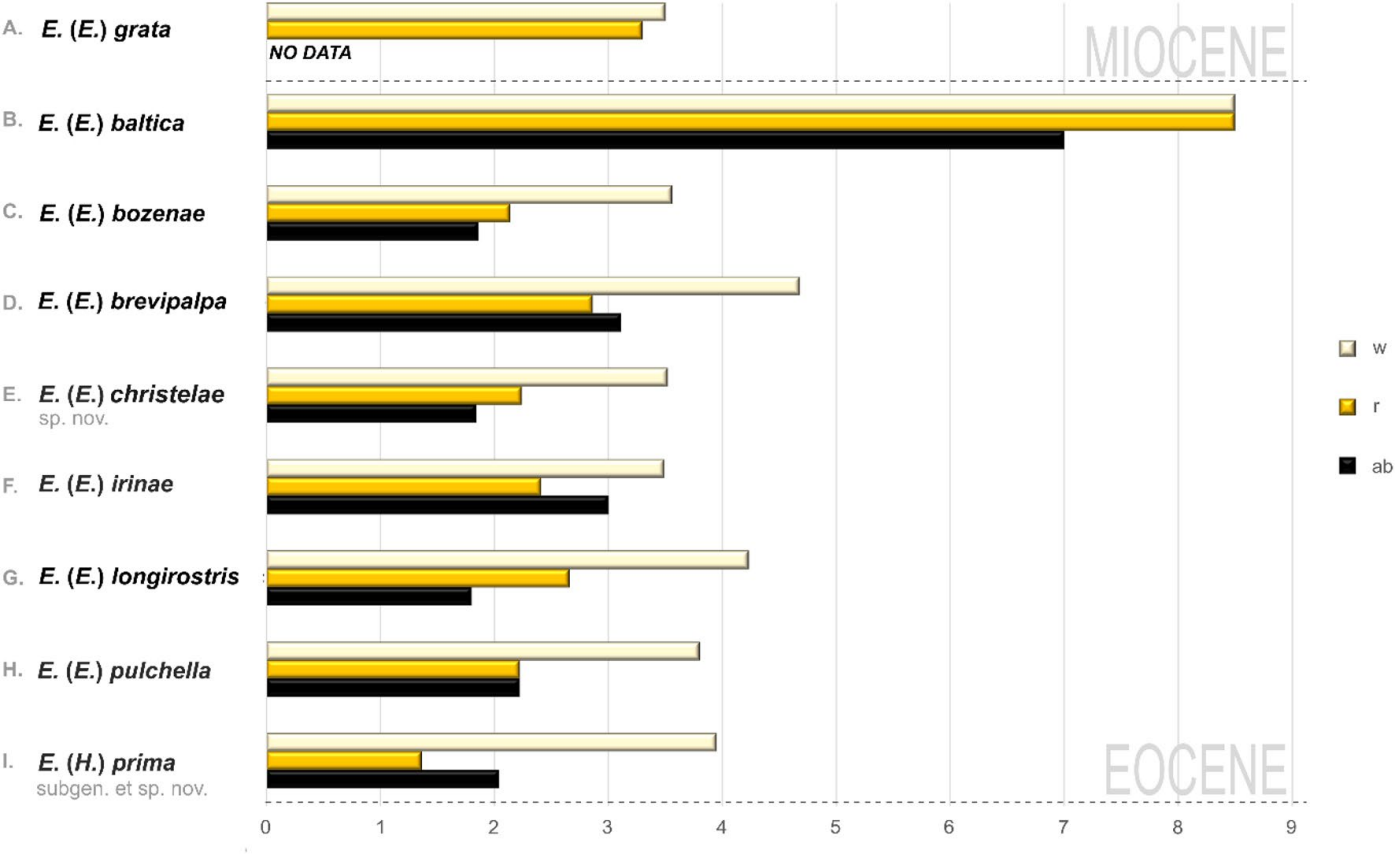
Relation between the length of wing (w), rostrum (r) and abdomen (ab) in different fossil species of *Elephantomyia*: (**A**) *Elephantomyia* (s. str.) *grata*, (**B**) *Elephantomyia* (s. str.) *baltica*, (**C**) *Elephantomyia* (s. str.) *bozenae*, (**D**) *Elephantomyia* (s. str.) *brevipalpa*, (**E**) *Elephantomyia* (s. str.) *christelae* sp. nov., (**F**) *Elephantomyia* (s. str.) *irinae*, (**G**) *Elephantomyia* (s. str.) *longirostris*, (**H**) *Elephantomyia* (s. str.) *pulchella*, (**I**) *Elephantomyia* (Hoffeinsonia) *prima* subgen. et sp. nov.

1. Rostrum longer than half wing length (Fig. 10A–H); tip of Sc before or at fork of Mb, before crossvein m-cu measured from the base of the wing; vein R_1_ at least slightly curved at the tip; vein R_2+3+4_ with a slight curve to the upper edge of wing, the distance between veins R_2+3+4_/R_1_ and veins R_2+3+4_/R_5_ comparable (Fig. 9A); gonostyles elongate, about 1/2 of the length of gonocoxite (Fig. 2F); ………………………………………..…. 2.
2. Rostrum measures approximately 1/3 of wing length (Fig. 10I); tip of Sc far beyond fork of Mb measured from the base of the wing, opposite crossvein m-cu ; vein R_1_ straight; basal half of vein R_2+3+4_ sharply arched to the upper edge of wing; veins R_2+3+4_ and R_1_ closer together than veins R_2+3+4_ and R_5_ (Fig. 9D); gonostyles small, about 1/3 length of gonocoxite (Fig. 5C); ………………..…..... ***E.* (*Hoffeinsonia*) *prima*** subgen et sp. nov
3. Veins M1+2 and M3 widely separated; the distance between veins M1+2/M3 and veins M3/M4 comparable; cross-vein m-m well developed (Fig. 8A–H) …………………... 3.
4. Veins M_1+2_ and M_3_ narrowly separated; the distance between veins M_1+2_/M_3_ smaller than between veins M_3_/M_4_; cross-vein m-m very short (Figs. 2E, 8I) ……………….… ………………………………..………………………..… ***E.* (s. str.) *christelae*** sp. nov.
5. Wings longer than rostrum (Fig. 10A, C–I); relatively short vein Rs, the length of vein Rs at least three times of the basal deflection of R_5_ …..……………………………….. 4.
6. Wings as long as rostrum (Fig. 10B); the length Rs only slightly longer than twice the length of the basal deflection of R_5_ (Fig. 8C) ................................................................ ………………………………….…..……………. ***E.* (s. str.) *baltica*** Alexander^6^
7. Palpus longer than the glossal lobes; antennae 15-segmented; Rs distinctly shorter than R2+3+4 ………………………………...………………….……………………………. 5.
8. Palpus shorter than one half of the glossal lobes of the rostrum; antennae 14-segmented; Rs as long as R_2+3+4_ or slightly longer (Fig. 8F) ………………………… ……………………………….……………………. ***E.* (s. str.) *brevipalpa*** Loew^7^.
9. D-cell distinctly elongate, narrow, approximately twice times as long as wide; vein M3 as long as d-cell …………………………………………………………………………………………6.
10. D-cell wide, approximetely1.5 times as long as wide; vein M3 1.5 times longer than d-cell …..………………………………………………………………………………… 7.
11. Fork of Rb far beyond half the length of wing, in 3/5 the length of wing measured from the base of wing; Rs short, approximately as long as d-cell; gonocoxite at least three times as long as wide (Fig. 8A) ……………………………………………………….. …………………………………………………..… ***E.*** **(s. str.)** ***grata*** Podenas & Poinar^9^
12. Fork of Rb before half the length of wing or just beyond, measured from the base of wing; Rs at least 1.5 × as long as d-cell; gonocoxite at most twice as long as wide (Fig. 8A) ………………………………………………………… ***E.*** **(s. str.)** ***bozenae*** Kania^8^.
13. Rostrum not very elongate, shorter than abdomen, distinctly shorter than wing, only slightly longer than 1/2 of wing length (Fig. 10E) …………………………………..…. ……………………………………………………………….. ***E.*** **(s. str.)** ***irinae*** Kania^8^
14. ………………………………………….. *E.* *pulchella* ^7,^ Rostrum elongate, as long or longer than abdomen, only slightly shorter than wing …. ……………………………………………………………………………………...…8.
15.  Wing approximately 1/3 longer than rostrum (Fig. 10H); cross-vein m-cu in 1/2 of d-cell length …………...…………………………….. ***E.*** **(s. str.)** ***pulchella*** (Loew, 1851)^7^
16. Rostrum very elongate, wing approximately 1/5 longer than rostrum (Fig. 10G); cross-vein m-cu shortly beyond the fork of Mb on M1+2 and M3+4 measured from the base of the wing (Fig. 8E) …………………………..…… ***E.*** **(s. str.)** ***longirostris*** Loew^7^.

## Discussion

Craneflies from the genus *Elephantomyia* to present were represented in the fossil record only by the subgenus *Elephantomyia*. The most well known species from the fossil record were described on the basis of inclusions in Eocene Baltic amber. We do not know the older representatives of the genus *Elephantomyia*. One species was described from a younger period (the Miocene)^9^ (Fig. 8) and to this day it has not been classified under any subgenus of the genus *Elephantomyia*. After careful analysis, it was possible to indicate herein that this species, like the other species described based on fossil material, belongs to the nominative subgenus *Elephantomyia. *So, of the four known subgenera of the genus *Elephantomyia*, only one is represented in the fossil record. The subgenus *Elephantomyia* is also the most diverse in modern fauna^5^.

There are over 100 species belonging to the subgenus *Elephantomyia* occurring in modern fauna. Subgenera like *Elephantomyina* and *Xenoelephantomyia* are represented by only single species^5^.

The characteristic morphological features of the newly discovered specimens mentioned in this paper allows the indication and description of a new subgenus of *Elephantomyia*.

The general features which characterize the genus *Elephantomyia* are: very elongate rostrum, sometimes longer than the body length, maxillary palpus four segmented, first palpomere reduced, atrophy of cross-vein r-r (R_2_) and comparatively short and wide gonocoxites with small gonostyles^25,29–31^. The combination of features such as legs at least partially pale, these parts almost white, vein R_1_ is straight, basal half of vein R_2+3+4_ is sharply arched to the upper edge of wing, veins R_2+3+4_ and R_1_ are positioned closer together than veins R_2+3+4_ and R_5_ and pale brown oval pterostigma, wing without colour pattern distinguish the new subgenus from other subgenera within the genus (Fig. 8).

It is also worth noting that the fossil subgenus *Hoffeinsonia* subgen. nov. shares features with the extant subgenera *Elephantomyodes* [such as basal half of vein R_2+3+4_ sharpy arched to the upper edge of wing, veins R_2+3+4_ and R_1_ running closer together than veins R_2+3+4_ and R_5_ and straight R_1_, reduced palpi, legs with very light (almost white) parts)] and *Elephantomyia* (such us oval pterostigma and wing distinctly wider than in *Elephantomyodes*) (Fig. 9). These similarities can indicate a phylogenetic relationship of *Hoffeinsonia* subgen. nov. and *Elephantomyodes* or *Elephantomyia*. The colour patterns of legs are rather rare in craneflies belonging to subgenus *Elephantomyia* and are very variable among taxa, eg. *Elephantomyia* (s. str.) *catarractes* Gavryushin 2016^32^ described from Tanzania, Morogoro env., Uluguru Mts, Majiyanakwendo waterfall, characterized by the colour pattern of legs which are mostly brown with tips of femora conspicuously yellow^32^. The relation between the length of wing, rostrum and abdomen of *Elephantomyia* (*Hoffeinsonia*) *prima* subgen. nov. is also different than in fossil representatives of *Elephantomyia*. The rostrum of *Elephantomyia* (*Hoffeinsonia*) *prima* subgen. et sp. nov. measures no more than 1/3 wing length, while in fossil *Elephantomyia* rostrum measures at least half wing length (Fig. 10).

Fossil records prove the existence of these insects as early as the Eocene. The discovery of the new subgenus sheds new light on the diversity of the genus *Elephantomyia*, the evolution of the Limoniidae and introduces important information that can be used in further research on the phylogenetic relationships of this group of insects.

The presence in Baltic amber of inclusions of flies of the genus *Elephantomyia* with a very elongate rostrum adapted to a specific food spectrum (most likely the nectar of Angiospermae flowers) can provide evidence that in “Baltic amber forests” were plants pollinated by these insects. This co-evolution of Angiospermae and Limoniinae began much earlier, in Cretaceous. From Cretaceous period are known species of genus *Helius* which are characterized by an elongate rostrum – flies adapted to feed on the nectar of Angiospermae flowers. This genus is closely related to *Elephantomyia*^33,34^.

It has been also proven that the Baltic amber flora comprises elements of both extant northern American and East Asian warm-temperature flora in “Baltic amber forests” and humid climate^20^. Recent species of subgenus *Elephantomyia* occur mainly in Neotropical and Afrotropical regions^3^ and are represented as an inclusions in Baltic amber while *Elephantomyodes* occur in Holarctic region. But, its absence in Baltic amber may be due to the fact that these insects were very rare in the Eocene, as in modern fauna^5^.

## Material and methods

The study was based on four inclusions in Eocene Baltic amber from the collection of Christel and Hans Werner Hoffeins. The holotypes of new described species herein are deposited in Senckenberg Deutsches Entomologisches Institut (SDEI) Müncheberg, Germany. The specimens were examined using a Nikon SMZ 1500 stereomicroscope equipped with a Nikon DS-Fi1 camera. The measurements were taken with NIS-Elements D 3.0 software. The length of head was measured as length of head capsule excluding rostrum. The length of the discal cell—measurements were given from its posterior edge to the point of connection of vein m-m with vein M_3_, the length of the vein M_3_—measurements were given from the point of connection of vein m-m with vein M_3_ to the margin of wing. The length of hypopygium was measured from the posterior margin of tergite IX to the apex of gonocoxite. The measurements and the relationship between the length of rostrum, wing and abdomen were given only in case when relevant structures were not distorted. Drawings were made by tracing the specimens and the photographs. Drawings and photographs (Figs. 2, 3, 4, 5, and 6, graphics 8–10 partially) were made by Iwona Kania-Kłosok. The map was built using the map Maps-For-Free (https:// maps- for- free. com) and modified with the software programs Corel Draw and Corel Photopaint X7. The stratigraphic chart was used according to International Stratigraphic Chart, International Commission of Stratigraphy (v. 2021/05) https://stratigraphy.org/chart. The wing venation follows that of^38^, terminology applied to the male genitalia nomenclature, is in accordance^8,30,39^.
