# Author Correction: Recent discoveries of new *Elephantomyia* (Diptera, Limoniidae) fossils in Baltic amber

**DOI:** 10.1038/s41598-021-04120-y

**Published:** 2021-12-21

**Authors:** Iwona Kania-Kłosok, Wiesław Krzemiński

**Affiliations:** 1 Department of Biology, Institute of Biology and Biotechnology, University of Rzeszów, Rzeszów, Poland; 2 Institute of Systematics and Evolution of Animals, Polish Academy of Sciences, Krakow, Poland

Correction to: *Scientific Reports*, 10.1038/s41598-021-03022-3, published online 8 December 2021

The original version of this Article contained an error in Affiliation 2, which was incorrectly given as ‘Institute of Systematics and Evolution of Animals, Polish Academy of Sciences, Warsaw, Poland’. The correct affiliation is listed below:

Institute of Systematics and Evolution of Animals, Polish Academy of Sciences, Krakow, Poland

The original Article has been corrected.

